# Generation and Genetic Correction of USH2A c.2299delG Mutation in Patient-Derived Induced Pluripotent Stem Cells

**DOI:** 10.3390/genes12060805

**Published:** 2021-05-25

**Authors:** Xuezhong Liu, Justin Lillywhite, Wenliang Zhu, Zaohua Huang, Anna M Clark, Nicholas Gosstola, Colin T. Maguire, Derek Dykxhoorn, Zheng-Yi Chen, Jun Yang

**Affiliations:** 1 Department of Otolaryngology, University of Miami Miller School of Medicine, Miami, FL 33136, USA; zxh359@med.miami.edu (Z.H.); ncg65@med.miami.edu (N.G.); 2 John P. Hussman Institute for Human Genomics, University of Miami Miller School of Medicine, Miami, FL 33136, USA; DDykxhoorn@med.miami.edu; 3 Interdisciplinary Stem Cell Institute, University of Miami Miller School of Medicine, Miami, FL 33136, USA; 4 Cellular Translational Research Core, Center for Clinical & Translational Science, University of Utah, Salt Lake City, UT 84112, USA; lillykvit@gmail.com (J.L.); colin.maguire@utah.edu (C.T.M.); 5 Department of Otolaryngology and Head-and Neck Surgery, and Program in Neuroscience, Harvard Medical School and Eaton Peabody Laboratory, Massachusetts Eye and Ear Infirmary, Boston, MA 02114, USA; Wenliang_Zhu@MEEI.HARVARD.EDU; 6 Department of Ophthalmology and Visual Sciences, John A. Moran Eye Center, University of Utah, Salt Lake City, UT 84132, USA; anna.clark@hsc.utah.edu

**Keywords:** patient-derived induced pluripotent stem cells, CRISPR/Cas9 gene therapy, USH2A

## Abstract

Usher syndrome (USH) is the leading cause of inherited combined hearing and vision loss. As an autosomal recessive trait, it affects 15,000 people in the United States alone and is responsible for ~21% of inherited blindness and 3 to 6% of early childhood deafness. Approximately 2/3 of the patients with Usher syndrome suffer from USH2, of whom 85% have mutations in the USH2A gene. Patients affected by USH2 suffer from congenital bilateral progressive sensorineural hearing loss and retinitis pigmentosa which leads to progressive loss of vision. To study the molecular mechanisms of this disease and develop a gene therapy strategy, we generated human induced pluripotent stem cells (iPSCs) from peripheral blood mononuclear cells (PBMCs) obtained from a patient carrying compound heterozygous variants of USH2A c.2299delG and c.1256G>T and the patient’s healthy sibling. The pluripotency and stability were confirmed by pluripotency cell specific marker expression and molecular karyotyping. Subsequent CRISPR/Cas9 genome editing using a homology repair template was used to successfully correct the USH2A c.2299delG mutation back to normal c.2299G in the generated patient iPSCs to create an isogenic pair of lines. Importantly, this manuscript describes the first use of the recombinant Cas9 and synthetic gRNA ribonucleoprotein complex approach to correct the USH2A c.2299delG without additional genetic effects in patient-derived iPSCs, an approach that is amenable for therapeutic genome editing. This work lays a solid foundation for future ex vivo and in vivo gene therapy investigations and these patient’s iPSCs also provide an unlimited resource for disease modeling and mechanistic studies.

## 1. Introduction

Usher syndrome (USH) is a devastating, clinically and genetically heterogenous disorder characterized by a triad of clinical phenotypes—sensorineural deafness, vision loss, and vestibular dysfunction [1,2]. With a combined prevalence of over 400,000 cases worldwide, USH represent the most common cause of deaf blindness (https://www.usher-syndrome.org/what-is-usher-syndrome/ (accessed on 5/5/2020)). Usher syndrome type II (USH2) is the most prevalent clinical form of USH, accounting for approximately two-thirds of USH patients. USH2 patients display less severe congenital hearing loss than USH1 patients and normal vestibular function. USH2 has three identified genomic loci that correspond to three distinct genes: *USH2A* (Usherin), *USH2C* (ADGRV1), and *USH2D* (Whirlin). Furthermore, *USH2A* mutations are responsible for up to 85% of USH2 [3,4]. Currently, 1518 variants have been found in the *USH2A* gene of which over 700 mutations are pathogenic. These variants are distributed throughout the entire gene (http://www.lovd.nl/USH2A (accessed on 5 May 2020)). The most common mutation in humans is the c.2299delG mutation that accounts for 55–90% of USH2 patients depending on the population being studied [3,5,6,7,8,9,10,11,12,13]. Genome editing technologies using programmable nucleases can modify the genome at a targeted locus and are recognized to be a valuable tool in studying the function of genes associated with hearing loss [14,15]. One important application of stem cells for deafness treatment is to use patient-derived induced pluripotent stem cells (iPSCs) to model the impact of specific genetic variants on disease pathogenesis. This approach helps in deciphering underlying pathogenic mechanisms and testing cutting edge gene editing methods including CRISPR/Cas9 to develop treatment modalities for the identified genetic defect [16,17,18]. Several human iPSCs have been generated from different USH2A mutation-bearing patient cells. These human iPSCs cover the following mutations: (c.949C > A; p.Tyr318Cysfs*17) and (c.1256G > T; p.Cys419Phe) compound mutation; (c.2299delG; p.Glu767Serfs*21) and (c.2276G > T; p.Cys759Phe) compound mutation; (c.2209C > T; p.Arg737Ter) and (c.8693A > C; p.Tyr2898Ser) compound mutation; (c.12575G > A; p.Arg4192His) heterozygous mutation; (c.2299delG; p.Glu767Serfs*21) homozygous mutation; and (c.2276G > T; p.Cys759Phe) homozygous mutation [19,20,21,22,23,24,25]. In addition, CRISPR-Cas9 genome editing has been used to correct USH2A (c.2299delG; p.Glu767Serfs*21) and (c.2276G > T; p.Cys759Phe) mutations using Cas9-sgRNA plasmid co-transfection with homology repair template [26]. In the current study, we generated a patient-derived iPSC line carrying c.2299delG mutation and successfully corrected this mutation using CRISPR/Cas9-mediated homology-directed repair (HDR). Compared to previous reports showing USH2A human iPSC generation and gene editing, this project has multiple unique innovations: 1. The generation of patient-derived human iPSCs with pathogenic, compound heterozygous USH2A variants (c.1256G > T; p.Cys419Phe) and (c.2299delG; p.Glu767Serfs*21), 2. The human iPSCs were derived from patient’s PBMCs rather than the more common dermal fibroblast-derived lines, 3. Recombinant Cas9-synthetic gRNAs complexes were used for the variant correction providing a more therapeutically relevant genome-editing approach compared to plasmid or viral-based systems. These vector-based approaches have increased potential for off-target effects and insertional mutagenesis compared to the use of the recombinant Cas9 and synthetic guide RNAs and templates [26]. The advantages of using PBMCs for the derivation of iPSC include the ease of blood collection, PBMC extraction, storage, and processing for downstream applications, while skin punch biopsies are more invasive with potential complications from infection, bleeding, and scaring. The isolation of fibroblasts is more labor-intensive. The benefits of Cas9-synthetic gRNA ribonucleoprotein include high efficiency, high specificity, easy standardization, and no insertional mutagenesis. The protocol can be easily quantified and standardized among different biomedical labs and institutions.

## 2. Materials and Methods

### 2.1. Patient Information

This study was approved by the University of Utah Institutional Review Board. A signed informed-consent form was collected from each participant. A female USH2 patient carrying a compound mutation of c.2299delG and c.1256G > T in the *USH2A* gene was recruited along with her healthy biological brother. Since both samples were from the same family and similar genetic background, the brother’s sample served as the best control for the patient sample. DNA sequences at mutation sites of both human subjects were sequenced to confirm the genetic mutations. The patient has a vision of OD 20/30; OS 20/30. She has a restricted periphery.

### 2.2. Generation of Patient-Derived Induced Pluripotent Stem Cells

For both human subjects, peripheral blood mononuclear cells (PBMCs) were isolated by density centrifugation using Histopaque-1077 (MilliporeSigma Corporate, St. Louis, MO, USA). The isolated PBMCs were expanded using Stem Pro-34 SFM media (Thermo Fisher Scientific, Waltham, MA, USA) with cytokines of SCF (100 ng/mL), IL-3 (10 ng/mL), EPO (2 units/mL), IGF-1 (40 ng/mL), and dexamethasone (1 µM). The expanded PBMCs were transduced by Sendai virus from the CytoTune ™-iPS 2.0 Sendai Reprogramming Kit (ThermoFisher Scientific) for reprogramming into induced pluripotent stem cells (iPSCs). At 3 days post transduction, the reprogrammed PBMCs were transferred onto vitronectin (RHVTN-N, ThermoFisher) coated wells (3.75 µg in 1.5 mL DPBS per well of a 6-well plate) and cultured in Stem Pro-34 SFM media. After 4 days, the reprogrammed cells were switched into TeSR™-E8™ medium (STEMCELL Technologies, Cambridge, MA, USA) without any supplement. Individual iPSC clones were picked and passaged for cell expansion in TeSR™-E8™ medium. Cultured cells were maintained at 37 ℃ and a 5% CO_2_ condition in an incubator, and the media were changed daily.

### 2.3. Reverse Transcription (RT) and Quantitative Polymerase Chain Reaction (qPCR) of Blood Cell Marker and Stem Cell Specific Genes

The pluripotency of reprogrammed iPSCs was checked by RT-qPCR. Human blood cell markers of CCR7, CD3D, CD8A, and CD4 were examined for their expression change after reprogramming. Pluripotent cell markers of NANOG, POU5F1 (OCT4), SOX2, and TFCP2L1 were tested for the success of iPSC generation. Total RNA was extracted from iPSCs and PBMCs using the PureLink RNA mini kit (ThermoFisher Scientific) and amplified by RT using the SuperScript VILO cDNA synthesis kit1 (ThermoFisher Scientific). After RT, 1 to 100 ng cDNA was used as the template for real time PCR using the QuantStudio 12K Flex system (Applied Biosystems, Foster City, CA). The main thermal cycler condition was 95 °C for 10 min, 40 cycles of 95 °C for 15 s, and 60 °C for 1 min. The real-time PCR results were analyzed by the QuantStudio 12K Flex software (version 1.2.2.). GAPDH (Glyceraldehyde-3-Phosphate Dehydrogenase) and RPLPO (Ribosomal Protein Lateral Stalk Subunit P0) genes were used as references. ΔΔCt was calculated between human PBMCs and reprogrammed iPSCs. Pre-validated proprietary primers from ThermoFisher Scientific were used for each reaction. Genes and assay IDs were as follows: GAPDH, Hs02786624; RPLPO, Hs00420895; CCR7, Hs01013469; CD3D, Hs00174158; CD8A, Hs00233520; CD4, Hs01058407; NANOG, Hs02387400; POU5F1, Hs04260367; SOX2, Hs01053049; and TFCP2L1, Hs01011666.

### 2.4. Immunostaining of Stem Cell Protein Markers and Trilineage Differentiation

Immunostaining was conducted to detect nuclear pluripotent stem cell markers of OCT4 and SOX2 and membrane pluripotent stem cell markers of SSEA-4 and TRA1-60. The medium was aspirated, and human iPSC clone cells were washed once with phosphate buffered saline (PBS) and fixed in 4% formaldehyde for 15 min, followed by three PBS washes. For membrane markers, the cells were blocked with 10% goat serum. For nuclear markers, the cells were permeabilized with 0.2% Triton X-100 and blocked with 10% goat serum. Cells were blocked or permeabilized for 40 min at room temperature. The incubation with primary antibodies was performed overnight at 4 °C and the incubation with secondary antibodies was performed at room temperature for 1 h. The primary antibodies used are listed as follows: rabbit monoclonal anti-OCT4 (1:200) (Cell Signaling Technology, Danvers, MA, USA), rabbit monoclonal anti-SOX2 (1:100) (Cell Signaling Technology), mouse monoclonal anti-TRA1-60 (1:200) (Cell Signaling Technology), and mouse monoclonal anti-SSEA-4 (1:100) (STEMCELL Technologies). The secondary antibodies were Alexa fluor 488 conjugated goat anti-rabbit (1:500) (Invitrogen, Carlsbad, CA, USA) and goat anti-mouse (1:500) antibodies (Invitrogen). Cell nuclei were counterstained with DAPI (Thermo Fisher Scientific). All images were taken using a Nikon inverted fluorescence microscope. Generated iPSCs were differentiated using the STEMDiff™ Trilineage Differentiation Kit from StemCell Technologies following the manufacturer’s instructions. The cells were plated at recommended densities and cultured with the lineage-specific media of the kit. At the end of the differentiation, differentiated cells were stained for specific germ layer markers using immunostaining: SOX17 for endoderm, brachyury for mesoderm, and βIII-tubulin for ectoderm. The primary antibodies used in germ layer markers detection were as follows: mouse monoclonal anti-SOX17 (1:100) (Abcam, Cambridge, MA, USA), rabbit polyclonal anti-Brachyury (1:200) (Abcam, Cambridge, MA), and mouse monoclonal anti-β-Tubulin III (1:100) (Sigma-Aldrich, St. Louis, MO, USA).

### 2.5. Molecular Karyotype Using a Nanostring Molecular Probe Array

Genomic DNA from human iPSCs was isolated using a PureGene DNA extraction kit (AutoGen Inc., Holliston, MA, USA). The concentration of DNA samples was measured using a Nanodrop spectrophotometer (ThermoFisher Scientific). For molecular karyotyping, the DNA samples were characterized using an nCounter Human Karyotyping Panel with Nanostring nCounter Analysis System (NanoString Technologies, Inc., Seattle, WA, USA). In brief, the genomic DNA of human iPSCs were fragmented with restriction endonuclease AluI, hybridized, and examined by bar coded probes of 338 individual loci in 24 human chromosomes. A normal karyotype sample was used as a reference to compare chromosome numbers. The data were analyzed using Nanostring nSolver 4.0 software.

### 2.6. Human iPSC Line Authentication

Short tandem repeat (STR) analysis was performed for cell line authentication. Twenty-four loci of human iPSC DNA were amplified using a Promega GenePrint 24 kit (Promega, Madison, WI, USA). These amplified targets were examined using an Applied Biosystems capillary electrophoresis system. The electropherogram results were analyzed and exported to a CSV file using GeneMarker HID software. Data in the CSV file were imported to the DSMZ database (Leibniz Institute DSMZ-German Collection of Microorganisms and Cell Cultures) to match the profile against other deposited cell lines.

### 2.7. Mycoplasma and Bacteria Detection

For mycoplasma testing, human iPSC DNA was examined using an e-Myco PLUS Mycoplasma PCR Detection Kit (iNtRON Biotechnology, Korea). The assay detects all important species, i.e., 8 genus and 209 species of mycoplasma. For bacterial testing, human iPSC DNA was examined using a PCR Bacteria Test Kit (PromoCell, Heidelberg, Germany).

### 2.8. Single Guide RNA Design, CRISPR/Cas9 Treatment of Patient iPSCs, and Cell Cloning

Single guide RNA (gRNA), AATTCTGCAATCCTCACTCT with a PAM sequence of GGG, was designed at the proximity of mutation site c.2299 by bioinformatic tools on the University of California, Santa Cruz genome browser and chemically synthesized by Synthego. The ribonucleoprotein (RNP) complex of Cas9 and sgRNA was prepared by incubating Cas9 protein (Integrated DNA technologies, Coralville, Iowa, 1.5 μM) and sgRNA (2 μM) at room temperature for 15 min. The sequence of the repair homology template was 5′ TAATGATGTTGGATGTGAGCCCTGCCAGTGTAACCTCCATGGCTCAGTGAACAAATTCTGCAATCCTCACTCTGGACAGTGTGAGTGCAAAAAAGAAGCCAAAGGACTTCAGTGTGACACCTGCAGAGAAAACTTTTATGGGTTAGATGTCAC 3′ (Integrated DNA Technologies).

Nucleofections were performed as previously described [27], patient-derived iPSCs were cultured until reaching 70~80% confluence on vitronectin coated 6-well plates in E8 medium. One hour before nucleofection, 10 μM ROCK- inhibitor Y27632 were added to the medium, then cells were dissociated by Accutase (Thermo Fisher A1110501) at 37 °C for 6–8 min and collected by centrifugation at 900 rpm for 3 min. Cells were counted, then washed by PBS and collected by centrifugation at 900 rpm for 3 min. Then, 0.2~0.4 million cells were resuspended in 20 μL P3 reagent, P3 Primary Cell 4D-Nucleofector^TM^ X Kit L (Lonza, Basel, Switzerland) with RNP. Next, 4 μM ssODN was used for a single nucleofection event and cells were nucleofected by the program CM-119 in Lonza nucleofector 4D (Lonza, Basel, Switzerland).

After nucleofection, 50% of iPSCs (0.1~0.2 million) were plated on a well of a vitronectin pre-coated 24-well plate in E8 medium. The other 50% iPSCs were plated on 6-well plates at low density (2000~20,000 cells per well). Then, 10 μM Y27632 were used for the first 24 h and gradually reduced to 1 μM over the next 3 days. Seventy-two hours after the nucleofection, cells in the 24-well plate were collected and genomic DNA was extracted by DNeasy kit (Qiagen, Germantown, MD, USA). PCR reactions were conducted with primers flanking the mutation site: forward primer (USH2AdelF, GGTGTGATCATTGCAATTTTGG) and reverse primer (USH2AdelR, CCCTGTCTTAGCATTACAGACAGTC). The PCR products were extracted using a PCR purification kit (Qiagen) and sent for next generation sequencing (NGS). Ten to fourteen days after the nucleofection, single colonies were picked from the 6-well plate and expanded. Genomic DNA was collected and PCR reactions were conducted and sequenced using the Sanger method.

### 2.9. Off-Target Analysis of CRISPR/Cas9 at Top Potential Genomic Loci

Top off-target sites were identified using the Synthego CRISPR Design Tool. Sequence specific primers were designed to generate PCR products covering the potential off-target sequences. PCR products were generated using genomic DNA extracted from parental cells and CRISPR/Cas9-treated single cell clones using DNeasy kit (Qiagen). All PCR samples were sent for Sanger sequencing after confirmation by agarose gel electrophoresis. The sequences were aligned between CRISPR/Cas9-treated samples and parental line samples to identify potential indels at the predicted sites. The primers (from 5′ to 3′) for the five top off-target sites were as follows: OFF1, forward, GTCTATATAACTCCCTTCTTTCAGG, reverse, CCTTTTAAGGTGACCTGGCAGCTG; OFF2, forward, GAACATGATAATATATGAACTCAAC, reverse, TCTGCAGTCTCCTCAGCCTCACTG; OFF3, forward, TGGGAAAGTTAAGCCGAGAGAAAG, reverse, AAGGCCACAGAGAAACAGATGAAAG; OFF4, forward, CTTCTGTCCCCAGTAGGGATGGTG, reverse, GGCAGGGCTCATGCAGAAGAGTTG; OFF5, forward, CAGAAGAGTTGCACCACATAGTTG, reverse, GTTCCTTCACCCAACATTTAAGTG.

## 3. Results

### 3.1. Generation of Patient-Derived Induced Pluripotent Stem Cells

**Figure 1:**
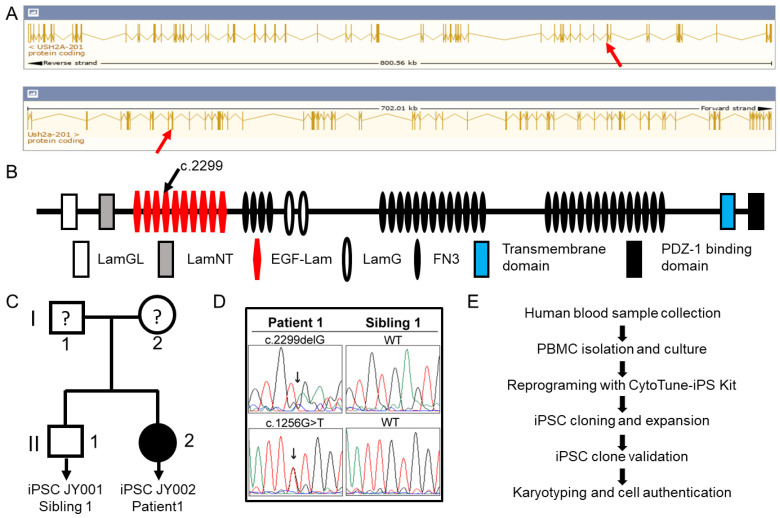
***USH2A* genomic structure, protein domain schema, pedigree information of human iPSC lines, and cell reprogramming.** (**A**) Genomic structure of the human and mouse *USH2A* gene. A frequent mutation of c.2299delG is marked by a red arrow in the human gene (top) and mouse gene (bottom) (www.ensembl.org (accessed on 5 May 2020)). (**B**) Protein domain structure of the human USH2A. The c.2299delG mutation location is marked by a black arrow above the domain; (**C**) We generated two human PBMC-derived iPSCs, labeled with JY001 and JY002. Line 1, JY001, was a health control; line 2, JY002, was diagnosed as USH2. JY001 and JY002 are siblings as indicated. The “?” mark stands for unknown phenotype and genotype. (**D**) The sequencing chromatograms of genetic mutation sites of patient 1 are presented along with those of her unaffected sibling. (**E**) Procedural flowchart of human PBMC reprogramming with four transcription factors.

The *USH2A* gene is one of the largest genes in both human and mouse and this gene is highly conserved. As indicated in Figure 1, the human *USH2A* gene spans ~800 Kbp and is composed 72 exons producing a transcript of 18,938 bps encoding a protein of 5202 amino acids. The mouse gene spans about 702 Kbp, has 71 exons and a transcript of 15,695 bps encoding a protein of 5193 amino acids. Human and mice share 71.44% amino acid identity. The USH2A proteins of human and mouse share a similar structural organization, which includes a large extracellular domain composed of LamGL, LamNT, EGF-Lam, LamG, and FN3 motif repeats, a membrane-spanning domain, and an intracellular PDZ-binding C terminus (Figure 1) [28]. As indicated in Figure 1, we have recruited a female USH2 patient bearing compound heterozygous c.2299delG and c.1256G > T *USH2A* variants (patient 1), together with a healthy biological brother as the unaffected control (sibling 1). After receiving their consent, whole blood was obtained using venipuncture and peripheral blood mononuclear cells (PBMCs) isolated by density gradient centrifugation. PBMCs were transduced with integration-free Sendai virus for reprogramming into induced pluripotent stem cells as shown in Figure 1. Clonal iPSC lines were generated for the affected individual (JY002) and the unaffected sibling (JY001), which were validated using several approaches.

### 3.2. Validation of Generated Human iPSCs Using RT-qPCR and Immunostaining

**Figure 2:**
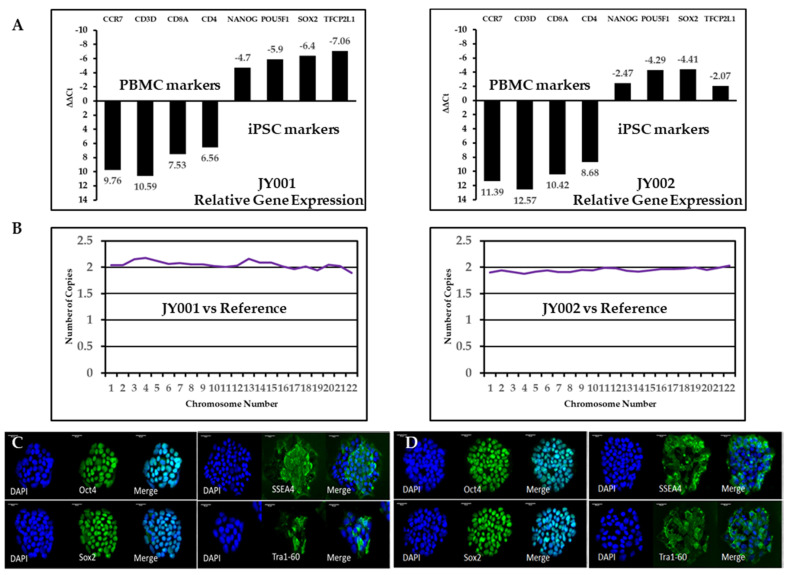
**Validation of iPSC lines.** (**A**) RT-PCR analysis. In both iPSC lines, pluripotent cell markers of *NANOG, POU5F1 (OCT4), SOX2,* and *TFCP2L1* are upregulated; on the other hand, blood cell markers of *CCR7, CD3D, CD8A,* and *CD4* are greatly reduced. (**B**) Molecular karyotyping. To screen for aneuploidy, we performed molecular karyotyping for all human chromosomes. Both iPSC lines had the expected number of copies for auto chromosomes (22 pair). Sex chromosome data are not shown here. (**C**) Normal sibling-derived human iPSC and (**D**) patient-derived iPSC carrying the c.2299delG mutation were stained with the nuclear pluripotent stem cell markers of OCT4, SOX2, SSEA4, and TRA1-60. Cell nuclei were counterstained with DAPI. The scale bar is 50 µm in all panels.

First, we applied reverse transcription (RT) and quantitative real time PCR to examine the specific gene expression profiles of these iPSC lines. We selected blood cell-specific markers CCR7, CD3D, CD8A, and CD4 and stem cell-specific markers POU5F1/OCT4, NANOG, SOX2, and TFCP2L1 to validate the successful reprogramming of these lines. As expected, expression of the blood-cell-specific markers were significantly reduced in the iPSC compared to those in the PBMCs: for JY001, CCR7 (0.0012 fold), CD3D (0.00065 fold), CD8A (0.0054 fold), CD4 (0.011 fold); for JY002, CCR7 (0.00037 fold), CD3D (0.00016 fold), CD8A (0.00073 fold), CD4 (0.0024 fold). On the other hand, expression of the stem cell-specific markers was significantly upregulated in the iPSC lines compared to that of the original PBMCs: for JY 001, POU5F1/OCT4 (25.99 fold), NANOG (59.71 fold), SOX2 (84.45 fold), TFCP2L1 (133.44 fold); for JY002, POU5F1/OCT4 (5.54 fold), NANOG (19.56 fold), SOX2 (21.26 fold), TFCP2L1 (4.20 fold). To corroborate our RT-qPCR results, immunocytochemical (ICC) analysis was performed by staining the iPSC lines using antibodies specific for stem/progenitor cell markers. These iPSC lines stained positively for the nuclear markers OCT4 and SOX2 and for the cell surface markers TRA-1-60 and SSEA4 (Figure 2C,D). The nuclear markers OCT4 and SOX2 co-localize with the nuclei stained with DAPI while the membrane markers were localized to the cell surface of the iPSC lines. The iPSCs were differentiated into the three primary germ layers using the Trilineage differentiation Kit and stained for the germ layer specific markers. The Supplementary Figure S1 shows the staining of the JY002 iPSC line that was differentiated using the Trilineage kit, showing positive staining for βIII-tubulin expression in the ectoderm differentiation, SOX17 in the endodermal differentiation, and brachyury in the mesoderm differentiation.

### 3.3. Cell Molecular Karyotyping and Authentication

The reprogramming process and subsequent culturing of the iPSC lines carries with them the potential for the introduction of chromosomal abnormalities. Therefore, a molecular karyotyping was performed using the Nanostring Human Karyotyping Panel. Cells known to have a normal karyotype were used as a reference in this experiment. This analysis showed that each iPSC line—JY001 and JY002—had a normal karyotype with no evidence of insertions or deletions within the detectable range of the assay. Sex chromosome profiles were also normal: JY001 is XY and JY002 is XX.

Short tandem repeat (STR) profiling was used to verify the identity of the PBMC-derived iPSC lines. This approach is a well-established human cell line authentication method due to its wide application in forensics identification. The iPSC lines JY001 (Table 1) and JY002 (Table 2) were compared to a panel of ten cell lines deposited in a large consortium of databases (DSMZ, ATCC, JCRB, and RIKEN). Table 1 and 2 shows the unique pattern of markers at the nine loci (D5S818, D13S317, D7S820, D16S539, VWA, TH01, TPOX, CSF1PO, AM (Amelogenin)). The marker pattern confirmed the sex of each sample using the AM (Amelogenion) marker. The matched repeats were labeled in red. In addition, these lines were shown to be free of mycoplasma and bacterial contamination as indicated in Supplementary Figure S2.

### 3.4. CRISPR/Cas9-Mediated Homology-Directed Repair and Single-Cell Cloning of Patient iPSCs

**Figure 3:**
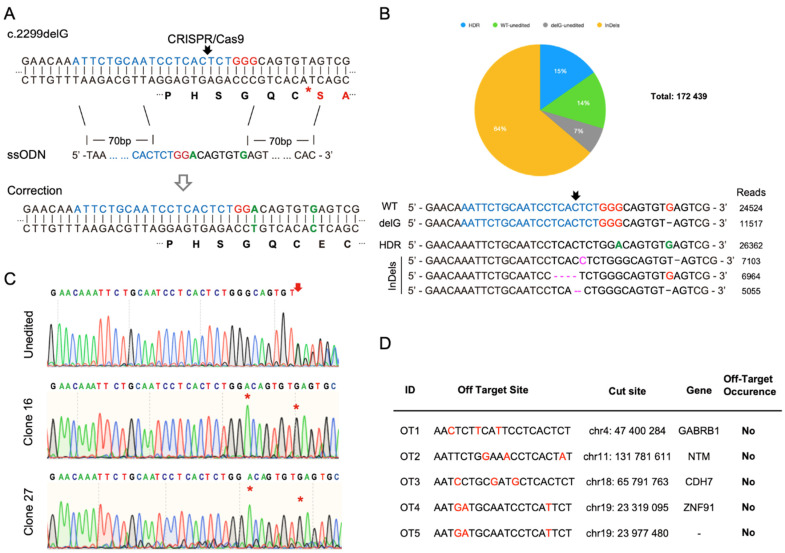
**Analysis of CRISPR/Cas9-mediated homology-directed repair for *USH2A* 2299delG mutation.** (**A**) Schematic outline of genetic correction using gene editing. Patient-derived *USH2A* c.2299delG iPSCs were treated with CRISPR/Cas9 reagents and a single strand homologous repair template. The 20 nt sgRNA targeting site is indicated in blue and the PAM site is indicated in red. Repaired bases in ssODN are indicated in green. The red asterisk indicates the delG mutation site. (**B**) Analysis of the next generation sequencing (NGS) results and demonstration of representative sequence reads for *USH2A* c.2299delG iPSCs after genetic correction. The gRNA sequence is indicated in blue and the PAM site in red. Repaired bases are indicated in green. InDels are indicated in magenta. (**C**) Sanger sequences of unedited *USH2A* c.2299delG iPSCs and clone 16, 27 after HDR genetic correction. Genomic DNAs of different clones were extracted for PCR using specific primers flanking c.2299delG. Red arrowhead indicates the delG mutation site and red asterisks indicate the G that was added back as well as a G-to-A flip in the PAM site. (**D**) Off-target study of CRISPR/Cas9-mediated homology-directed repair of c.2299delG mutation. The top potential off-target sites of our gRNA are listed, and the mismatched base is marked in red.

CRISPR/Cas9 genome editing and HDR were used to correct the c.2299delG mutation in our patient-derived iPSC line. Small guide RNAs (sgRNA) were designed using sequencing information derived from the University of California, Santa Cruz (UCSC) genome browser and are shown in Figure 3A. We applied a 153 bp ssODN (single stranded donor oligonucleotides) as the homology repair template in which the G was added back and flanked by two 70 bp homology arms. To avoid unwanted indels (insertion/deletion) after genetic correction, a G-to-A change was also introduced in the ssODN to disrupt the protospacer adjacent motif (PAM), without affecting the amino acid sequence, as shown in Figure 3A. To mimic clinic conditions and reduce off-target effects, we used a recombinant Cas9 and synthetic sgRNA ribonucleoprotein (RNP) complex approach to treat the patient-derived iPSCs. Three-day post-CRISPR/Cas9-mediated HDR treatment, gene editing efficiency was measured using next generation sequencing (NGS) and was shown to have a 79% cutting efficiency with 15% of reads showing correction of the deletion (i.e., proper HDR of the deletion) (Figure 3B).

Single CRISP-edited iPSC clones were derived by seeding individual iPSC cells into a 96-well plate following RNP treatment. The cells expanded into distinct colonies, which were manually collected and propagated. Forty-five individual clonal lines were expanded. Genomic DNA was extracted, the region around the deletion PCR was amplified, and the resulting product was Sanger sequenced to identify clonal iPSC lines in which the deletion had been corrected. Among these clonal iPSC lines, 2 clones (clone #16 and #27) were identified to have corrected the deletion and modified the PAM sequence without any additional alterations (i.e., incorporation of insertions or deletions (indels)) (Figure 3C). In addition, the top five potential off-target sites were identified using Synthego CRISPR Design Tool and these regions were PCR amplified in the deletion-corrected iPSC clones and sent for Sanger sequencing to ensure that no off-target events occurred during the CRISPR/Cas9-mediated genome editing. As indicated in the off-target data table in Figure 3D, we did not detect any off-target effects of the CRISPR treatment in these loci in either of the deletion-corrected iPSC lines.

### 3.5. Bioinformatic Analysis of Utilizing a Mouse Model for a Human USH2A c.2299delG Study

**Figure 4:**
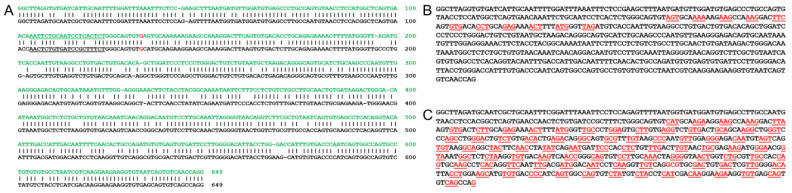
**Comparison of human and mouse *USH2A* c.2299 exon sequences.** (**A**) Human *USH2A* exon 13 DNA in green carrying c.2299G are aligned with mouse exon 12 DNA in black carrying c.2299G with gRNA underlined. The sequence alignment can help to develop the CRISPR/Cas9 gene editing strategies and predict outcomes of using mouse as in vivo models. (**B**) Human *USH2A* c.2299delG are listed to show the early termination of protein translation. (**C**) However, mouse *Ush2a* c.2299delG does not result in early termination of the protein translation. This sequence analysis can help to develop the CRISPR/Cas9 gene editing strategies and predict outcomes of using mouse as in vivo models.

Finally, we wanted to explore the feasibility of utilizing a mouse model for a human *USH2A* c.2299delG mutation study. When we examined closely the mouse *Ush2a* gene structure of exon 12, which is a counterpart of the human *USH2A* gene exon 13 that contains c.2299, the gRNA site remained as shown in Figure 4A. However, the consequences of c.2299delG are totally different in human *USH2A* and mouse *Ush2a* genes. For the human *USH2A* gene, c.2299delG results in an early protein translation termination as shown in Figure 4B (stop codon TAG in red); while for the mouse *Ush2a* gene, c.2299delG does not result in an early termination and the entire exon can be read through as seen in Figure 4C. Therefore, whether mouse is a good model for a human *USH2A* c.2299delG study needs to be addressed cautiously. Potential solutions could be to prepare a humanized mouse at the c.2299 site by replacing mouse exon 12 with human exon 13 or simply introducing a point mutation to change the codon TGC into TGA for the same early termination as in humans.

## 4. Discussion

Induced pluripotent stem cell (iPSC) technology is a powerful tool that provides a way to model the impact of genetic variants on disease pathogenesis in disease-relevant cells. This approach was first developed over a decade ago by the work of Yamanaka and colleagues and was quickly adopted by many labs [29,30]. Patient-derived iPSCs now play critical roles in disease modeling, drug development, organ generation, and therapeutic studies [31]. The finding and optimization of the clustered regularly interspaced short palindromic repeats (CRISPR)/CRISPR-associated (Cas) protein 9 system, an original bacterial adaptive immune system, has revolutionized the genome-editing field [32,33,34]. For genetic correction, a repair DNA template, a double strand DNA or a single strand DNA, as short as 100 bp, is provided that contains the desired nucleotide change to trigger the homology-directed repair (HDR) pathway. However, this pathway is significantly less efficient than is non-homologous end joining. The combination of patient-derived iPSCs and CRISPR/Cas9 genome editing can provide critical disease models for gene therapy investigations.

In this study, we generated patient-derived iPSCs from an individual bearing a compound heterozygous mutation in USH2A, including the most common mutation of *USH2A*: c.2299delG. Since these variants in USH2A act in an autosomal recessive manner, correction of only one of the alleles would be sufficient to restore the wild-type functionality. This iPSC line offers unlimited material for disease modeling and therapeutic studies of *USH2A* c.2299delG disorders. In the hearing loss field, patient-derived iPSCs could be differentiated into inner ear organoids and multiple cell types, including hair cell-like cells, spiral ganglion cells, and Schwann cells for therapeutic and mechanism studies [17,35,36,37,38,39,40,41,42]. Furthermore, the variant-corrected iPSCs can be utilized for precise medicine for USH2 patients without immunological rejections when being used for cellular transplantation and differentiation. There is no evidence to suggest that genome editing in iPSC alters their pluripotent phenotype. In fact, our results support other studies that showed that iPSC lines retain their pluripotent nature and differentiation potential following CRISPR-mediated genome editing [26,43]. We utilized CRISPR/Cas9-mediated HDR to correct the c.2299delG mutation with high efficiency, according to NGS analysis, but with a relatively low accuracy of 4% when individual genome edited iPSC clones were tested. There may be a couple of explanations for why differences were seen by NGS analysis of the pooled-cells and single-cell cloning: (1) The targeting site limits our choice of sgRNAs, as the only available sgRNA cutting site is 12 bp away from the mutation. In general, a cutting site 10 bp or less away from a PAM is recommended [44,45]. Nevertheless, we proved our sgRNA was efficient enough to correct the mutation in patient iPSCs. (2) The mutant site was not within the sgRNA region, hence CRISPR/Cas9 may cut both the mutant and the wild-type alleles. The corrected colonies we identified were all homozygous HDR repaired products. (3) Although we mutated the PAM site with a synonymous point mutation in the repair template, there might still be re-cutting of the fragment by Cas9/gRNA, as indels could still be identified on the alleles after repair in NGS results. Change of GGG into XGG (X is A, C, or T) or GGX (X is A, C, or T) might also cause Cas9 to re-cut since the the PAM site NGG remains in both situations and Cas9 is known to tolerate such mismatches [45]. An alternative is to use a novel less error prone prime editing with pegRNA (prime editing guide RNA) [46]. The prime editing involves a novel Cas9 nickase fused to a reverse transcriptase and a long pegRNA that encodes the repair template RNA in addition to the gRNA. This system utilizes reverse transcription to introduce the desired DNA repairs in the cell genomic DNA at specific sites, including deletions, insertions, and point mutations.

Although USH2A c.2299delG iPSC generation and correction has been previously published, this project has multiple novelties: we generated patient-derived human iPSCs with the novel c.1256G > T (p.Cys419Phe) in addition to the more common c.2299delG (p.Glu767Serfs*21) variant; the derived iPSCs were from patient PBMCs rather than fibroblasts or keratinocytes, making this approach more patient-friendly while reducing the barrier to patient enrollment in studies; and instead of using plasmid-based genome editing tools, we used the ribonucleoprotein (RNP) approach, reducing the possibility of plasmid DNA integration into the cell genome and reducing the potential off-target effects. As for the impact of the correction of c.2299delG on the compound mutation, the literature suggests that one normal USH2A allele is necessary for the normal cochlea and retinal function [47], and heterozygous c.1256G > T seems to have no known pathological effect [48]. It is expected that the c.2299delG-corrected iPSC will restore normal functionality.

In summary, we generated a unique *USH2A* compound mutation iPSC line and a healthy control line from an unaffected sibling. These lines provide valuable resources and materials for *USH2A* disease mechanism studies, drug screening, and therapy development. In addition, we established a protocol to correct the c.2299delG mutation in the patient-derived iPSCs. In future, we will test if the genetic correction could rescue the phenotype of the mutation in the auditory and visual systems using series comparison studies between mutated and corrected iPSCs. The corrected iPSCs can be further explored for in vivo cell-based therapies, reducing the obstacles for in vivo delivery and differentiation into auditory and visual system cells. Our work on correcting the *USH2A* variants supports the use of this approach to test the effect of numerous variants in a variety of deafness genes using a single normal human iPSC cell line. These isogenic iPSC lines could then be differentiated into cells from the inner ear or more complex 3D inner ear organoids to disease phenotypes and therapeutic strategies.

## Figures and Tables

**Table 1:** Short tandem repeat profiling of JY001.

EV	Cell No.	Cell Name	Locus Names								
			D5S818	D13S317	D7S820	D16S539	VWA	TH01	AM	TPOX	CSF1PO
	JY001		11, 11	8, 12	10, 11	11, 13	15, 18	9.3, 9.3	x, y	11, 11	10, 12
0.78 (28/36)	CRL-2529	CCD1124Sk	11, 12	8, 12	10, 12	11, 13	15, 18	7, 9.3	X, Y	8, 11	10, 12
0.67 (24/36)	557	ROS-50	11, 11	8, 13	10, 11	8, 12	17, 18	6, 9.3	X, Y	11, 11	11, 12
0.67 (24/36)	563	HCC-78	11, 11	8, 12	9, 9	11, 13	15, 18	7, 9.3	X, Y	8, 11	11, 12
0.67 (24/36)	740	HD-MB03	11, 11	11, 12	9, 10	9, 11	18, 19	6, 9.3	X, Y	8, 11	10, 12
0.67 (24/36)	CRL-2096	CCD-1076Sk	11, 11	8, 11	10, 11	11, 13	16, 18	6, 9.3	X, Y	8, 8	11, 12
0.67 (24/36)	CRL-5882	NCI-H1648[H1648]	11, 11	12, 12	10, 11	11, 11	14, 17	7, 9.3	X, Y	8, 11	10, 12
0.67 (24/36)	CRL-5964	NCI-BL2077	11, 11	8, 12	10, 12	12, 13	18, 20	8, 9.3	X, Y	8, 11	10, 11
0.67 (24/36)	CRL-7425	Hs 688(A)T	8, 11	12, 13	10, 11	9, 13	15, 18	7, 9	X, Y	8, 11	10, 12
0.67 (24/36)	CRL-7426	Hs 688(B)T	8, 11	12, 13	10, 11	9, 13	15, 18	7, 9	X, Y	8, 11	10, 12
0.67 (24/36)	CRL-7833	Hs 172T	11, 11	12, 12	8, 11	13, 13	18, 21	9.3, 9.3	X, Y	9, 11	11, 12

**Table 2:** Short tandem repeat profiling of JY002.

EV	Cell No.	Cell Name	Locus Names								
			D5S818	D13S317	D7S820	D16S539	VWA	TH01	AM	TPOX	CSF1PO
	JY002		11, 13	11, 12	10, 11	11, 12	14, 18	7, 9.3	x, x	8, 11	12, 12
0.78 (28/36)	468	SIG-M5	11, 13	11, 12	9, 9	11, 12	17, 19	7, 9.3	X, X	8, 11	12, 12
0.72 (26/36)	42	697	11, 13	11, 12	10, 11	11, 12	16, 18	8, 9	X, X	8, 11	11, 12
0.72 (26/36)	55	A-498	11, 13	12, 12	10, 11	12, 12	16, 18	6, 9.3	X, X	8, 11	11, 12
0.72 (26/36)	326	SW-948	11, 11	10, 11	9, 11	11, 12	16, 18	6, 9.3	X, X	8, 11	12, 12
0.72 (26/36)	CCL237	SW-948[SW-948]	11, 11	10, 11	9, 11	11, 12	16, 18	6, 9.3	X, X	8, 11	12, 12
0.72 (26/36)	CRL-1594	C-4I	9, 11	11, 12	10, 11	11, 11	14, 14	9.3, 9.3	X, X	10, 11	12, 12
0.72 (26/36)	CRL-1595	C-4II	9, 11	11, 12	10, 11	11, 11	14, 14	9, 9.3	X, X	10, 11	12, 12
0.72 (26/36)	CRL-1718	CCF-STTG1	12, 13	11, 13	10, 11	11, 12	17, 17	7, 8	X, X	8, 11	12, 12
0.72 (26/36)	CRL-7193	Hs 228.T	11, 12	8, 9	10, 11	11, 12	14, 18	8, 9.3	X, X	8, 11	11, 12
0.72 (26/36)	CRL-7242	Hs 329.T	11, 13	9, 11	11, 12	11, 12	17, 18	6, 9.3	X, X	8, 11	11, 12

## Supplementary Materials

The following are available online at www.mdpi.com/xxx/s1,s2, Figure S1: Trilineage differentiation of JY002; Figure S2: Contamination evaluations of iPSC cultures.
