# Supplementary material for: Generation and Genetic Correction of USH2A c.2299delG Mutation in Patient-Derived Induced Pluripotent Stem Cells
Source: Genes (Basel). 2021 May 25;12(6):805. doi: 10.3390/genes12060805 (PMC8227183; doi:10.3390/genes12060805)
Supplement: Supplementary file 1 [file genes-12-00805-s001.zip › genes-1214881-supplementary.pdf]

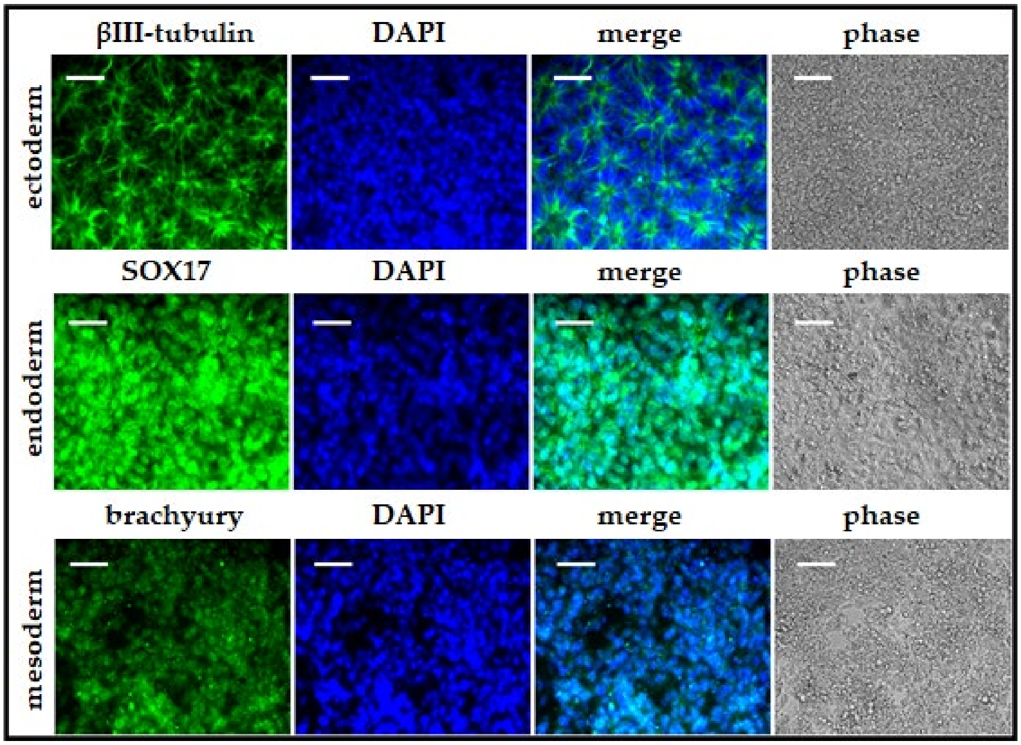

Figure S1. Trilineage differentiation of JY002. JY002 iPSCs were differentiated using the STEMDiff™ Trilineage Differentiation Kit from StemCell Technologies. The cells were plated at densities according to the manufacturer's instructions and cultured with lineage-specific media from the kit. At the end of the differentiation, differentiated cells were stained for specific germ layer markers as indicated: βIII-tubulin for ectoderm differentiation, SOX17 for endodermal differentiation, and brachyury for mesoderm differentiation. The scale bar is 50 μm.

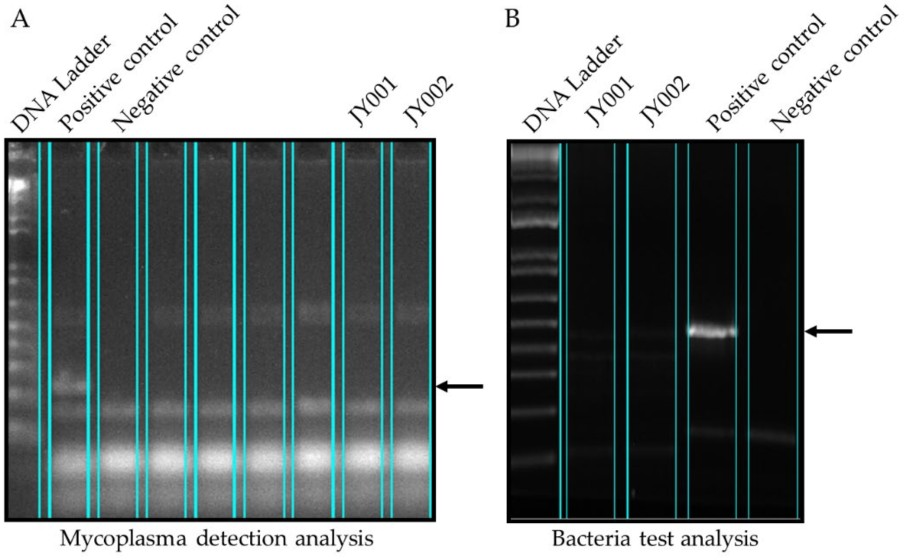

Figure S2. Contamination evaluations of iPSC cultures. (A) For mycoplasma detection analysis, JY001 and JY002 DNA were examined using an e-Myco PLUS Mycoplasma PCR Detection Kit. The assay detects all important mycoplasma species, i.e., 8 genus and 209 species of mycoplasma. Both iPSC cultures were not contaminated with mycoplasma. The right arrow points to the position of the positive band as indicated in the positive control sample. (B) For bacterial contamination testing, JY001 and JY002 DNA were examined using a PCR Bacteria Test Kit. No bacteria were detected in both iPSC samples. The right arrow points to the position of the positive band as indicated in the positive control sample.
